# Mutations in zinc finger 407 [*ZNF407*] cause a unique autosomal recessive cognitive impairment syndrome

**DOI:** 10.1186/1750-1172-9-80

**Published:** 2014-06-07

**Authors:** Marios Kambouris, Rachid C Maroun, Tawfeg Ben-Omran, Yasser Al-Sarraj, Khaoula Errafii, Rehab Ali, Hala Boulos, Patrick A Curmi, Hatem El-Shanti

**Affiliations:** 1 Qatar Biomedical Research Institute, Medical Genetics Center, 69 Lusail Street, West Bay Area, P.O. Box: 33123, Doha, Qatar; 2 Yale University School of Medicine, Genetics, New Haven, CT, USA; 3 Laboratoire Structure-Activité des Biomolécules Normales et Pathologiques, Institut National de la Santé et de la Recherche Médicale (INSERM), Université d’Evry Val d’Essonne, Evry 91025, France; 4 Clinical & Metabolic Genetics, Pediatrics, Hamad Medical Corporation, Doha, Qatar; 5 Weill Cornell Medical College, Doha, Qatar; 6 Shafallah Medical Genetics Center, Doha, Qatar; 7 University of Iowa, Pediatrics, Iowa City, IA, USA

**Keywords:** Zinc finger proteins, Cognitive impairment, Homozygosity mapping, Next generation exome sequencing, In-silico protein modeling

## Abstract

**Background:**

A consanguineous Arab family is affected by an apparently novel autosomal recessive disorder characterized by cognitive impairment, failure-to-thrive, hypotonia and dysmorphic features including bilateral ptosis and epicanthic folds, synophrys, midface hypoplasia, downturned mouth corners, thin upper vermillion border and prominent ears, bilateral 5th finger camptodactyly, bilateral short 4th metatarsal bones, and limited knee mobility bilaterally.

**Methods:**

The family was studied by homozygosity mapping, candidate gene mutation screening and whole Exome Next Generation Sequencing of a single affected member to identify the offending gene and mutation. The mutated gene product was studied by structural bioinformatics methods.

**Results:**

A damaging c.C5054G mutation affecting an evolutionary highly conserved amino acid p.S1685W was identified in the *ZNF407* gene at 18q23. The Serine to Tryptophane mutation affects two of the three ZNF407 isoforms and is located in the last third of the protein, in a linker peptide adjoining two zinc-finger domains. Structural analyses of this mutation shows disruption of an H-bond that locks the relative spatial position of the two fingers, leading to a higher flexibility of the linker and thus to a decreased probability of binding to the target DNA sequence essentially eliminating the functionality of downstream domains and interfering with the expression of various genes under ZNF407 control during fetal brain development.

**Conclusions:**

ZNF407 is a transcription factor with an essential role in brain development. When specific and limited in number homozygosity intervals exist that harbor the offending gene in consanguineous families, Whole Exome Sequencing of a single affected individual is an efficient approach to gene mapping and mutation identification.

## Background

A pregnant woman and her first cousin spouse, both of Qatari ethnic origin, presented for prenatal diagnosis because of family history of two previous male children with marked cognitive impairment and dysmorphic features, as well as, two early first trimester abortions. The cause of the miscarriages is unknown. To identify the gene and mutation responsible for the phenotype and to propose a possible underlying molecular mechanism, Homozygosity mapping, candidate gene screening, Next Generation Exome Sequencing and bioinformatics analyses of the resulting data were utilized.

## Methods

Homozygosity mapping was performed for all family members utilizing the Human Mapping 370 K-Cyto12 SNP genotyping array [Illumina, USA]. For the determination of the homozygosity intervals, data were analyzed by the HomozygosityMapper software [1].

Sanger sequencing using Big-dye terminator v.3.1 cycle sequencing [Applied Biosystems, USA] was performed on an ABI 3730 automatic sequencer [Applied Biosystems, USA] to screen for mutations in candidate genes, perform population frequency studies for variants and determine co-segregation of variants with the disease phenotype within the family.

Whole Exome target enrichment Next Generation Sequencing was performed on ABI SOLiD4 platform [Applied Biosystems] according to manufacturer’s specifications. DNA library preparation was with TargetSeq™ Exome Enrichment system [Applied Biosystems] as multiplex fragments libraries utilizing both the SOLiD^®^ Fragment Library Construction Kits and SOLiD^®^ Fragment Library Barcoding Kit Module 1–16 for the SOLiD^®^ 4 System. Bead preparation and enriching was done on an EZ Bead Emulsifier, Amplifier and Enricher utilizing E80 scale. Sequencing modality was with multiplex fragment paired-end.

Bioinformatic analyses of whole Exome NGS data was as follows: Raw data files (in a proprietary XSQ file format) were analyzed with the LifeTechnologies LifeScope v2.5.4 software running on a dedicated cluster to align the reads produced by the SoLID to a hg19 whole genome reference sequence, sourced from the University of California, Santa Cruz Genome Informatics Group (UCSC). The aligned BAM files were validated, duplicate sequences were identified and removed and incorrectly identified Mate-Pairs were corrected using the Picard v1.87 software. The Genome Analysis Tool Kit (GATK) v3.0.0 was applied to the ‘corrected’ output files to recalibrate the base quality scores, using machine learning to model any systematic errors in the data; carry out localized realignments around possible insertion/deletion sequences to ensure mapping accuracy; identify viable variants from the sequence reads; and recalibrate the variants to ensure accuracy of the variant calling, in a variant-type specific manner. Once a suitable list of variants was produced in this manner, the list was filtered using in-house scripts to confirm variant zygosity and identify those variants that conform to the inheritance model. These variants were annotated using an in-house script in conjunction with Annovar to produce an annotated list of variants with the most recent information available on a number of reference websites.

As the three-dimensional [3-D] structure of the ZNF407 protein is not available in the Protein Data Bank (PDB), sequence homology methods were utilized to construct 3-D models of the wild type and mutant zinc finger domains 18 and 19 of ZNF407. BLAST-searches (http://blast.ncbi.nlm.nih.gov/Blast.cgi) were performed within the PDB for crystal structures of homologous proteins. Once these were identified, the CLUSTALW software (http://www.ebi.ac.uk) was utilized to insure correct sequence alignments between domains 18 and 19 of ZNF407 and the retrieved zing finger structures found in the PDB. Focus was on complexes with double-stranded DNA only so as to have the proper bioactive conformation of the tandem finger. A high sequence homology with the Aart protein in complex with dsDNA (PDB code 2I13) was identified. The 3-D model of ZNF407 was then generated with the Modeller software (version 9v8) [2] based on the crystal structure of Aart in complex with DNA as a template (PDB code 2I13). A cycle of energy minimizations was then performed to obtain a stable conformation of the 3-D model. The quality of the model generated was finally assessed with DOPE score [3] and the Ramachandran plot of the best model was calculated by ProCheck [4] to discriminate unfavorable amino-acids backbone conformations. Three-Dimensional ribbon representations of the models were obtained with the molecular graphics program PyMOL (http://www.pymol.org). To generate the 3-D model of the mutant, Serine 1685 was replaced for a Tryptophan residue in the second finger of the 18–19 tandem finger domain and submitted to the same protocol as that used for the wild type complex. Thereafter both models were compared for an insight into the structural and thermodynamic stability effects of the mutation. To generate the 3-D model of the mutant, Serine 1685 was replaced for a Tryptophan residue in the second finger of the 18–19 tandem finger domain and submitted to the same protocol as that of the wild type complex. Thereafter, both models were compared for an insight into the structural and thermodynamic stability effects of the mutation.

The study was conducted in accordance with the provisions of the Declaration of Helsinki and an informed consent was obtained by the guardian of the affected participating family member.

## Results

### Clinical presentation

Individual II:2 is an 11-year-old boy who was born at full term after an uneventful pregnancy, labor and delivery. Although the birth growth parameters are not recorded, the parents recall that all measurements were within normal limits, including the head circumference. Currently, his height is 124 cm (below the 3^rd^%ile) and his weight is 27 kg (at the 3^rd^%ile) and is reported to have a normal head circumference. His development has been delayed; he sat without support at 3 years and walked independently, with an awkward gait and bent knees, between the age of 4 and 5 years. He started to talk with very limited speech at the age of 8 years and is not toilet trained till now. Evaluation by Leiter-R [Leiter International Performance Scale] indicated severe cognitive and developmental disability with a Brief IQ of 36. The physical examination showed bilateral ptosis and epicanthic folds, synophrys, strabismus, midface hypoplasia, downturned corners of the mouth, thin upper vermillion border and prominent ears with marked folding and absent lobules. He had bilateral short fourth metatarsal bones with overriding toes, bilateral proximal insertion of the thumbs and persistent fetal pads on fingers and toes. He is hypotonic with exaggerated deep tendon reflexes. The skeletal survey showed normal hips and mild kyphosis without scoliosis. Conventional karyotyping, Array CGH, Echocardiogram and abdominal ultrasound were all reported as normal. An MRI of the brain is normal, without any evidence of structural abnormalities. Hearing test (auditory evoked potential) has been reported to be normal.

**Figure 1:**
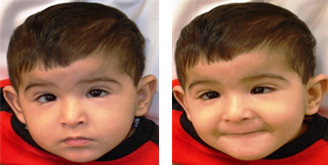
Dysmorphic features of affected individual II-4: Bilateral ptosis and epicanthic folds, synophrys, midface hypoplasia, downturned corners of the mouth, thin upper vermillion border and prominent ears.

Individual II:4 is a 5-year-old boy who was born at 37 weeks after an uneventful pregnancy, labor and delivery. His birth weight is 2.95 kg and length is 48 cm and head circumference of 33.5 cm, all are within normal limits. Currently, his height is 95 cm (below the 3^rd^%ile) and his weight is 15 kg (at the 3^rd^%ile) and is reported to have a normal head circumference. His development has been delayed; he currently sits without support and attempts to pull himself to a stand. He babbles infrequently and reaches for objects and is not toilet trained till now. Evaluation by Leiter-R [Leiter International Performance Scale] could not be performed, and the Vinland Adaptive Behavior Scale gave a total scaled score of 164 and an adaptive behavior total grade of 38, which denotes severe developmental and cognitive disability. The physical examination showed bilateral ptosis and epicanthic folds, synophrys, strabismus, midface hypoplasia, downturned corners of the mouth, thin upper vermillion border and prominent ears with marked over-folding and absent lobules (Figure 1). He has bilateral camptodactyly of the fifth finger, bilateral proximal insertion of the thumbs, overriding toes and persistent fetal pads on fingers and toes. He is hypotonic with exaggerated deep tendon reflexes. The skeletal survey showed bilateral femoral joint subluxation, dysplastic left acetabulum, and mild kyphosis without scoliosis. Conventional karyotyping, Array CGH, Echocardiogram and abdominal ultrasound were all reported as normal. An MRI of the brain is normal, without any evidence of structural abnormalities. The family history is remarkable for first cousin parents, two early spontaneous abortions and two normal siblings.

**Figure 2:**
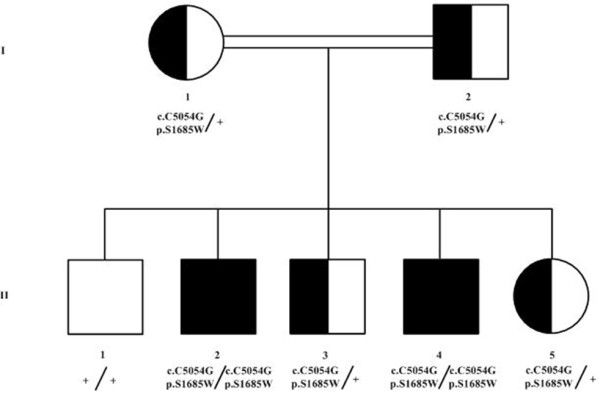
**Pedigree of the family segregating for the *ZNF407 *c.C5054G/p.S1685W mutation.** Genotypes are indicated. Consanguinity line represents first cousin marriage. Two early miscarriages are not shown. Half shaded blocks indicate genotypes only. Full shaded blocks indicate both genotypes and phenotypes for the affected individuals.

Homozygosity mapping was performed for all family members (Figure 2) except II:5. The mutation-harboring gene was mapped to four possible genome homozygosity intervals: Hsa 8p [rs1786342/101,676,363 to rs4336584/109,940,377, length 8.3 Mb], Hsa 14q [rs12431815/40,004,891 to rs1958628/48,107,640, length 8.1 Mb], Hsa 15q [rs12148268/58,759,459 to rs4886727/75,957,375, length 17.2 Mb], Hsa 18q [rs12454898/71,590,636 to rs11081575/77,707,525, length 6.1 Mb]. A screen of genes on these intervals, with respect to the clinical findings, suggested a possible positional candidate gene *MAP2K1* located on chromosome 15. The gene was screened by DNA Sanger sequencing for pathogenic mutations but none were identified. Furthermore, since both affected individuals are males, the genotyping data were analyzed for shared regions on the X-chromosome between the two affected males. One shared interval was identified [Hsa X, rs311183/2,724,756 to rs2681655/9,479,898, length 6.8 Mb]. Four positional candidate X-linked genes (taking into consideration the clinical findings): *PRKX*, *ARSF*, *ARSD* and *ARSH* were screened for mutations by DNA Sanger sequencing; again, no pathogenic mutations were identified. Whole Exome target enrichment Next Generation Sequencing of one of the affected individuals resulted in the identification of multiple exonic non-synonymous homozygous variants, with only five localizing in the four major homozygosity intervals. 1) *IGDCC4:* c.G727A/p.D243N; 2) *C15orf39*: g.G46293956A/p.R1029K; 3) *TLN2:* c.G6716A/p.R2239H; 4) *PML:* c.G1487T/p.G496V; 5) *ZNF407:* c.C5054G/p.S1685W.

DNA from individual II:5 (Figure 2), a fetus at the time, was obtained through amniocentesis and was genotyped genome-wide identical to all other family members as well as for the c.C5054G/p.S1685W mutation. The analyses indicated that II:5 is a carrier for the c.C5054G/p.S1685W mutation (heterozygous) and the genome-wide genotyping confirms heterozygosity for the critical interval on chromosome 18 containing *ZNF407*. At birth it was re-confirmed that individual II:5 was not affected.

## Discussion

Homozygosity mapping, screening of positional candidate genes, whole Exome Next Generation Sequencing and data mining were utilized in the effort to identify the disease causing gene and mutation for this apparently novel rare autosomal recessive disease. Out of the five potentially damaging variants identified within the homozygosity intervals, the *IGDCC4:* c.G727A/p.D243N [rs35223184]; and *C15orf39*: g.G46293956A/p.R1029K [rs149175372] were immediately excluded since they represent known common polymorphisms with >1% population frequency [1000 Genomes and NCBI dbSNP Build 135].

The third identified variant, *TLN2* [c.G6716A/p.R2239H], affects a non-conserved amino acid. The “alternate” amino-acid Histidine is found as the “normal” amino-acid at that position in numerous non-human species. A gene trap mouse model with disrupted *Taln2*, as well as *Taln2* knockout mice are viable and fertile but may display a mild dystrophic phenotype in skeletal and cardiac muscles [5-7] without any neurological findings as in the family under investigation. These render this variant as extremely unlikely pathogenic in relation to the clinical phenotype of the patients.

The fourth identified variant c.G1487T/p.G496V affects a non-conserved amino acid in a known Promyelocytic Leukemia tumor suppressor [*PML*] gene in Acute Promyelocytic Leukemia (APL). *PML* mutations have been detected in APL as well as in other hematopoietic malignancies. Functional loss of *PML* does not relate at all to the phenotype of the family [8], and again this variant is extremely unlikely to be pathogenic in relation to the phenotype under investigation.

The fifth variant, a c.C5054G/p.S1685W at the zinc finger transcription factor gene (*ZNF407*), was found to affect an evolutionarily highly conserved amino acid. It has damaging effects according to PolyPhen and SIFT protein-modeling software [9,10], and it co-segregates with the disease phenotype within the family. It is absent in any of the publically available variant databases [1000 Genomes and NCBI dbSNP Build 135]. Furthermore, it is absent in 400 ethnically matched control chromosomes, making it the only and most likely disease-causing gene and mutation.

Zinc finger genes are found ubiquitously, and categorized into several types based on the structure of their binding residues, and their biological roles within the cell [11,12]. Through their ability to bind to DNA, RNA & proteins, zinc finger proteins facilitate numerous cellular functions and processes including, but not limited to, the control of hematopoiesis, regulation of gene expression, cell differentiation, and development [13]. Their DNA-binding domain is comprised of tandem repeats of two, three or more fingers that can have different binding specificities. These tandem arrays, separated by amino acid linkers, bind through sequence-specific contact to 2–4 bases in the major groove of the DNA, spaced at 3-bp intervals [14,15]. Optimal binding is achieved through the sequential binding and wrapping of the zinc finger domains around the DNA [15].

The genomic sequence of *ZNF407*, as reported in the Uniprot database of proteins [Uniprot consortium, 2012] is 434,710 bp long and generates a 2,248 amino acid protein product of 247,367 Da. with 22 zinc finger domains. Each of the 22 zinc finger motifs comprises from 23 to 26 amino acid residues. The 22 zinc fingers come in tandems of one (fingers 6, 7, 12 and 13), two (fingers 4–5, 8–9, 10–11), three (1–3, 14–16) and six (17–22) fingers.

*ZNF407* constitutes one of the 481 ultra-conserved elements in the human genome, rendering it a functionally essential gene [16]. It presents three isoforms produced by alternative splicing. Isoform 1 (Q9C0G0-1) is the “canonical” sequence. Isoform 2 (Q9C0G0-2) differs from the canonical sequence in amino acids 1811–1815 and amino acids 1816–2248 are missing. In isoform 3 (Q9C0G0-3), amino acids 1625–1660 are modified and residues 1661–2248 are missing. This isoform thus does not possess zinc fingers 18 to 22.

The c.C5054G [p.S1685W] mutation affects two of the three *ZNF407* isoforms. It is located in the last third of the ZNF407 sequence and affects a Serine residue in the linker between zinc finger domains 18 and 19. These two zinc fingers belong to the 6-finger tandem comprising fingers 17–22. Zink fingers 18 and 19 represent amino acid residues F1656-H1680 and F1686-H1708, respectively, and are missing in isoform 3. Perturbation or modification of the linker adjoining two zinc-finger domains possibly result in the loss of DNA binding domains, thus eliminating their functionality and subsequently interfering with the expression of various genes under the zinc-finger gene control. Genes under ZNF407 control have not been as of yet identified.

**Figure 3:**
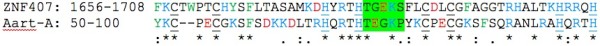
**CLUSTALW sequence alignment between zinc fingers 18 and 19 of ZNF407 with their corresponding linker (TGEKS, highlighted in green), and zinc fingers 2 and 3 of Aart and its linker (TEGKP, highlighted in green).** Asterisks denote sequence identity; colons, sequence similarity as defined by scores coming from the substitution matrix; and dots, sequences with any small positive scores. The residues whose side chains chelate the Zn^2+^ ion in Aart are underlined (C52, C55, H68, H72 for ZNF407, and C80, C83, H96, and H100 for Aart-A); the Serine residue that undergoes the mutation to Tryptophan is in bold. Basic residues are in blue and acidic in red.

**Figure 4:**
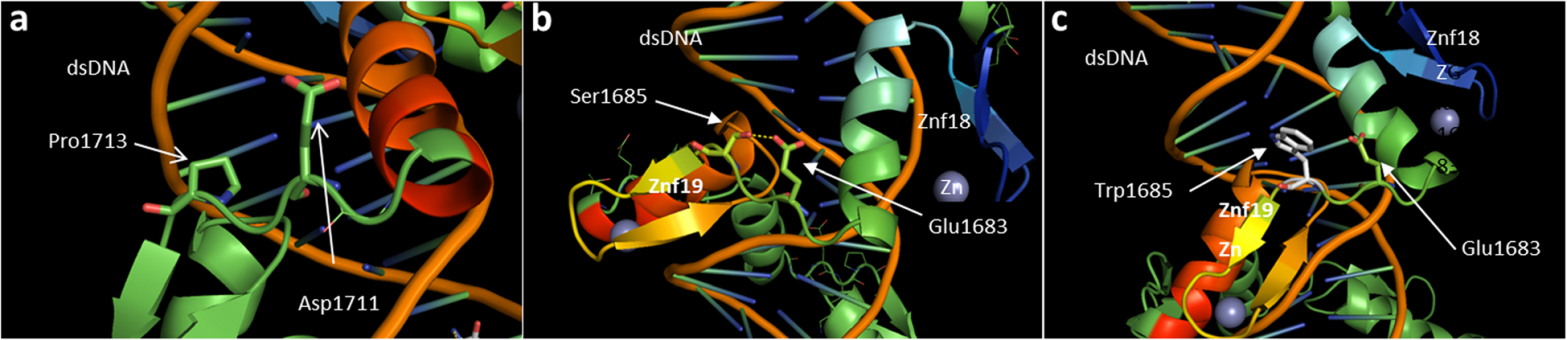
**Diagrams of the 3D model of ZNF407 tandem finger domains (rainbow colored) and a double-stranded DNA sequence (phosphate backbone in orange and bases as green-to-blue sticks). a**: Wild type finger 19-finger 20 linker with p.P1713 and p.E1711 (positions i + 4 and i + 2, respectively). **b**: Wild type fingers 18 and 19 with linker peptide showing p.S1685 H-bonded to p.E1683. One zinc ion is coordinated by each finger (grey spheres). **c**: p.S1685W mutant showing the loss of the H-bond to p.E1683.

The homology modeling approach is justified since the identity rate between the sequence of an unknown structure and a sequence whose experimentally available structure is high. Figure 3 shows the sequence alignment between fingers 18 and 19 of the target sequence (ZNF407), as annotated in UniProt, and the sequence corresponding to fingers 2 and 3 of the high resolution crystal structure (Protein Data Bank code 2I13, subunit A) of a tandem 6-finger zinc finger domain (Aart) bound to a double-stranded DNA and designed to recognize ANN triplets [17]. This alignment shows a high degree of identity between the two sequences (Figure 3). In spite of the 2-residue insertion in the N-terminus of zinc finger 18 of ZNF407 (Threonine-Tryptophan, Figure 3), the high sequence identity (~40%) between the two sequences allows us to model-build a 3D structure of the ZNF407-DNA complex in order to visualize the effects of the Serine to Tryptophan mutation. Since no specific DNA sequence for ZNF407 binding are available, the interaction of tandem fingers 18 and 19 with the DNA sequence was modeled after the Aart-DNA complex. Figure 4 shows diagrams of the 3D model of wild type ZNF407 tandem finger domains 18 and 19 wrapped around the major groove of the double-stranded DNA sequence. The zinc finger linkers are five residues long in general (residues i to i + 4) and usually contain a Proline residue at position i + 4. The Proline side chain is known for its rigidity because of its cyclic character. Its presence at position i + 4 thus restrains the conformation of the linker, reducing its flexibility, and properly positioning the α-helix of the C-terminal finger for interaction with the target DNA. This is illustrated in the case of fingers 19 and 20 (Figure 4a). Accordingly, by reducing the overall conformational entropy, complex formation is favored energetically and structurally.

The presence of Serine in position i + 4 of the *ZNF407*18-19 linker is unusual and carries with it added backbone flexibility. Nevertheless, this potential flexibility is reduced by an H-bond between the hydroxyl group of S1685 at i + 4 and the carboxyl side chain of E1683 at i + 2, locking the relative spatial position of the 18 and 19 fingers. This stabilizes the linker in a β-strand-like conformation and favors the interaction with DNA by reducing the number of bioactive conformations available for binding (Figure 4b). A Serine to Tryptophan mutation in the 18–19 zinc finger linker abolishes this H-bond, leading to a more flexible linker, and thus to a decrease in the probability of zinc finger-DNA complex formation of downstream partners (Figure 4c). The expected physiological effects of this physico-chemical perturbation include interfering with the expression of genes under ZNF407 control.

DNA- and RNA-binding zinc finger proteins are transcriptional regulators with a major function controlling developmental cascades of gene expression especially during fetal brain development. Mutations in such proteins have been found to interfere with normal brain development manifested during the developmental period, thus causing severe mental retardation. They have been associated with non-syndromic X-linked mental retardation [*ZNF81*: OMIM 314998 [18]; *ZNF674*: OMIM 300573 [19], *ZNF711*: OMIM 300803 [20], autosomal recessive non-syndromic intellectual disability [*ZC3H14*, OMIM 613279] [21] and impairment in adaptive behavior that manifest during development.

*ZNF407* mRNA, as well as the corresponding protein are expressed in most normal tissues with moderate nuclear and cytoplasmic positivity. These include adult, embryonic and fetal central and peripheral nervous systems (http://www.nextprot.org/db/entry/NX_Q9C0G0/expression). It is not known if there is tissue specificity for each of the three isoforms or what tissues express the two isoforms affected by the c.C5054G/p.S1685W mutation. To date, twenty polymorphisms for *ZNF407* have been reported in the Domain Mapping of Disease Mutations database (DMDM) [22]. The natural variants reported in the UniProt database entry Q9C0G0 (N69S, G512R, N972T, S1259L, A1913T) are included in DMDM. None of these, though, have been associated with known diseases. However, deletion of a critical region on 18q23 that includes the *ZNF407* gene and two other zinc finger genes (*ZNF516* and *ZNF236*), among other genes, is correlated with congenital aural atresia (CAA), which manifests a subset of phenotypes recognized by the 18q deletion (18q-) syndrome [23]. However, the clinical picture described in this syndrome is probably due to haploinsufficiency of several genes, as we could not find any symptomatic presentation in obligate carriers. Analysis of several CAA patients led to defining the breakpoint of the deletion within *ZNF407*[24]*.* Another identified translocation breakpoint in the third intron of the *ZNF407* gene causes a reduction in the transcript of its isoform 1, resulting in non-syndromic intellectual impairment and autism [25]. Furthermore, two *de novo* damaging missense mutations [c.A1436G/p.Y460C; c.C3640G/p.P1195A] in the very long linker region between zinc fingers 3 and 4, and between 11 and 12, respectively, of the *ZNF407* gene were found in one intellectual impairment patient each. Both patients were heterozygous, each for their relevant mutation.

This is the first association of *ZNF407* mutation to an autosomal recessive syndromic intellectual impairment/mental retardation detected using Whole Exome Sequencing of a single affected individual following the determination of specific gene mapping intervals through homozygosity mapping. This proved to be the most efficient approach to gene and mutation identification, as compared to multiple family-member Whole Exome Sequencing with the resulting comparative analyses, or to candidate gene screening by Sanger sequencing when multiple candidates exist.

Next Generation Exome and Genome sequencing have clearly demonstrated that humans harbor numerous deleterious mutations with no apparent ill effect [26,27]. The built-in redundancy in the human genome provides alternate pathways that make possible compensation for deleterious mutations in important genes without which there would be incompatibility with life. Thus, deleterious mutations resulting from NGS data need to be supported by additional genomic or functional data whenever possible. The *ZNF407* c.C5054G/p.S1685W mutation is the only mutation that lies within one of the homozygosity intervals that is certain to harbor the offending gene that could not be excluded as the disease causing mutation. It co-segregates with the disease phenotype within the family, it is not found as a polymorphic variant in publically available databases or in the ethnic population and it affects an evolutionarily highly conserved amino acid. Normal sequence variants [polymorphisms] have not been reported in *ZNF407* linker regions (between zinc fingers) providing further evidence of the sequence specificity required by these regions for normal function and subsequently for the damaging effects of the mutation. Extensive protein modeling clearly indicates that the mutation interferes with the normal function of the ZNF407 protein.

Taken together, these data highlight the significance of the c.C5054G/p.S1685W mutation and the role of the *ZNF407* gene in controlling the expression of downstream genes involved in pathways leading to normal fetal brain development. Once disrupted, one or more dimensions of brain development get affected. With this pausing as a pivotal step in normal brain development, it is worth understanding the underlying molecular mechanism by which this mutation eliminates or significantly alters the functionality of ZNF407, thus compromising the developmental pathway ZNF407 is involved in. The model presented proposes a rational account of such molecular mechanism.

## Competing interests

The authors declare that they have no competing interests.

## Authors’ contribution

MK and HE-S: Conception and design, project management and coordination, data interpretation, candidate gene selection, manuscript preparation, final approval of the version published. TB-O and RA: Patient Recruitment, Clinical assessment. PAC and RCM: Protein modeling. YA-S: Homozygosity mapping analyses, Next Generation Exome Sequencing, data acquisition and analyses. KE: Sanger Sequencing, candidate gene screening, variant validation, population screening, data acquisition and analysis. HB: Manuscript preparation, revision and submission coordination. All authors read and approved the final manuscript.
